# The paired *t*-test as a simple latent change score model

**DOI:** 10.3389/fpsyg.2013.00738

**Published:** 2013-10-10

**Authors:** Emil N. Coman, Katherine Picho, John J. McArdle, Victor Villagra, Lisa Dierker, Eugen Iordache

**Affiliations:** ^1^TRIPP Center, University of Connecticut Health CenterFarmington, CT, USA; ^2^Department of Medicine, Uniformed Services UniversityBethesda, MD, USA; ^3^Department of Psychology, University of Southern CaliforniaLos Angeles, CA, USA; ^4^Psychology Department, Wesleyan UniversityMiddletown, CT, USA; ^5^Forestry Department, Transilvania UniversityBrasov, Romania

**Keywords:** paired *t*-test, latent change score, statistical power, true changes, Structural Equation Modeling, introductory statistics

The *t*-test is a common statistical test of differences in means. Despite the fact that its extension, the paired *t*-test (*t*-test_P_), appears in most introductory statistics textbooks, it is less known that for repeated variables the *t*-test_P_ is in fact a model of change that can be replicated within the Structural Equation Modeling (SEM) framework. We show how to perform the *t*-test_P_ with latent change scores (LCS) models, which allow for direct testing of significance of mean changes, and moreover can explain inter-individual (and group) differences in changes over time.

## Modeling background

Structural models with latent variables can nowadays replicate virtually any statistical test (Skrondal and Rabe-Hesketh, 2004). It has been shown in particular that a latent growth curve model (LGM) with *df* = 0 fully replicates the *t*-test_P_ statistical tests for two waves of data (Voelkle, 2007) and yields asymptotically identical results. LCS models themselves fully replicate the repeated measures Anova and LGMs models and results, and additionally are much broader and capable to relax their assumptions (McArdle, 2009). We show how a more flexible LCS model with changes correlated with initial levels fully replicates the *t*-test_P_, and moreover can be expanded to test more complex hypotheses of change.

We demonstrate our proposal with a dataset on changes in Hemoglobin A1c (HgA1c) levels among diabetics who participated in a quality improvement study using peer supporters (Phase II–Diabetes eCo-System, Villagra, 2013). HgA1c values range between 5 and 12% (HgA1c > 6.5% defines a diabetic patient, NGSP, 2013). Many studies reporting changes in HgA1c values use the *t*-test_P_ to test for significance of changes (e.g., Satoh-Asahara et al., 2013). Hence, we tested the changes in HgA1c in a group of diabetics using the *t*-test_P_, and replicated these results in the LCS framework.

**Figure 1:**
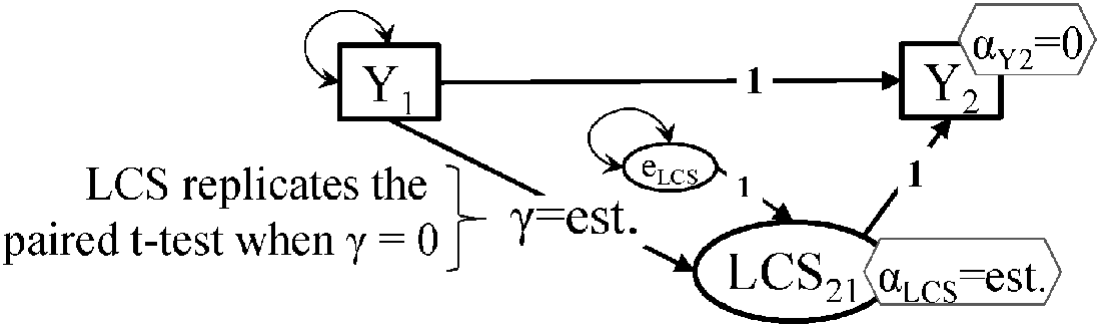
**Structural modeling setup for the paired *t*-test model and the latent change score (LCS) model with self-feedback/mediation (SF/M).** Notes: significance of *Y*_1_ to *Y*_2_ changes is captured by the α_LCS_ intercept; ‘est.’ means parameter is estimated by the model; intercepts are shown in hexagons, variances as double-headed arrows (covariance with itself); the key features of the LCS mode are: (1). No *Y*_2_ residual error; (2). Intercept of *Y*_2_ is set to zero; (3). The auto-regressive path is set to 1; the LCS to *Y*_2_ path is set to 1.

The key feature of the *t*-test_P_ is the computation of the sample average of differences (ΔY_21i_ = Y_2i_ − Y_1i_) which is compared to zero, using an estimate of its standard error that accounts for the covariance between initial levels and changes. Because the *t*-test_P_ is testing the significance of *Y*_1_ to *Y*_2_ changes, a natural alternative is to directly specify the change score (*Y*_2_ − Y_1_ or ΔY_21_) in a model of change. Because of known issues around the reliability of the change score (King et al., 2006) and estimation problems due to adding a variable that is a linear combination of two other variables in the same model, the LCS directly inserts the change score in a structural model, however, as a latent variable. Rewriting the ΔY_21_ = Y_2_ − Y_1_ equation as LCS_21_ = Y_2_ − Y_1_, one can define the true changes (McArdle and Nesselroade, 1994) with the multiple regression:

$$Y_{2}=0+1*Y_{1}+1*{\text{LCS}}_{21}+0$$

where we specifically indicate that the two regression coefficients are fixed to 1 (unity), and the intercept of *Y*_2_ is set to zero, as are the mean and variance of the *Y*_2_ residual error, i.e., the entire variance of *Y*_2_ is explained by the true change LCS_21_ (see Figure 1).

While *t*-test_p_ statistical outputs from various software report the correlation between the two repeated measures, the role of this indicator of measure stability is not directly obvious in the *t*-test_P_ model. There are two ways in which one can incorporate stability information into the LCS model of change: by specifying a correlation between *Y*_1_ and LCS_21_ (like McArdle, 2001; Figure 16.3), or with a directed path from *Y*_1_ to LCS_21_. There is a clear difference in terms of the test of significance of changes in outcome Y between the two setups: the correlation specification tests the significance of the mean of the LCS_21_ score and yields identical results with the *t*-test_P_, while the γ path specification is testing the significance of the LCS_21_ intercept; hence, differences in significance level will be observed.

The simple model in Figure 1 is asymptotically equivalent to the *t*-test_P_, when *Y*_1_ is correlated with LCS_21_, or when the γ path is set to zero (in the directed path setup). The γ path has been called proportional growth (McArdle, 2009), immanent change (Coleman, 1968), self-feedback (McArdle, 2009), or self-mediation (Coman et al., 2013); we label it here as self-feedback/mediation (SF/M). It can be easily shown that the γ coefficient's absolute value is the complement (1 - ρ) of the common auto-regressive (AR1) path, or stability (Kenny, 1979, p. 287), from a simple lagged model *Y*_1_ → *Y*_2_ (Coman et al., 2013): when γ = 0, there is perfect stability (ρ = 1). When an outcome changes in level but under perfect stability (equivalent to the LCS model with zero SF/M), the knowledge of one's initial outcome value *Y*_1_ means perfect prediction of his/her *Y*_2_ follow-up value; the *Y*_2_ intercept in this case tells us by how much Y increases (or decreases) uniformly across the entire sample, i.e., the change is the same in all persons/cases.

This particular case of perfectly stable outcomes is rarely found in real life. Change usually happens within some boundaries, and often those starting off higher on an outcome will change less. The likelihood of this hypothesis can be tested against the data however: since the more relaxed LCS model with SE/M path γ estimated has *df* = 0, directly forcing γ = 0 on the full LCS model tells us whether the assumption is reasonable or not: a significant χ^2^ of the no-SF/M LCS model, or equivalently: a significant worsening of fit Δχ ^2^(1), tells us whether the “no SF/M link” hypothesis is tenable.

## Results

We performed the *t*-test_P_ in MS Excel, then replicated it in the free AMOS 5 (Arbuckle, 2007) software, as a LCS model with *Y*_1_ and LCS_21_ correlated, then with *Y*_1_ predicting LCS_21_, then forcing γ = 0 (all our models and data are posted at http://trippcenter.uchc.edu/modeling). To be able to meaningfully compare the results, the outcomes were both centered on the HgA1c_1_ mean, and thus the HgA1c_2_ mean became −0.395. The *t*-test_P_ for significance of HgA1c changes in the diabetic patients (*n* = 97) indicates a mean of the paired differences of *d* = −0.395, *SE* = 0.182, *t* = −2.173, *p* = 0.032, and a paired sample correlation ρ = 0.566, *p* < 0.001. The LCS model with *Y*_1_ and LCS_21_ correlated yields similar results, whether the correlation is estimated or set to zero, *d* = −0.395, *SE* = 0.182, *CR* = −2.173 (critical ratio), *p* = 0.0297. The LCS with a SF/M path, however, yields a slightly lower *p*-value for the test of significance of changes, *d* = −0.395, *SE* = 0.171, *CR* = −2.307, *p* = 0.021. We note that the LCS model with no SF/M path (γ set to 0) indicated unacceptable misfit, χ ^2^(1) = 11.423, *p* = 0.001. This tells us that the path coefficient's γ = 0 constraint is not supported by data.

The difference between *p*-values from the models with the correlated initial level and changes vs. initial level predicting the changes occurs largely because the LCS model with SF/M path tests the significance of the HgA1c_2_ intercept, i.e., the mean changes *controlled for* HgA1c_1_.

To further illustrate our ideas, we re-tested the significance of the HgA1c reported in a recent study (Satoh-Asahara et al., 2013) for their control group (*n* = 24), from published summary data (means and variances), in the absence of reported information about the *Y*_1_ - *Y*_2_ correlation ρ. A re-test of the original classical paired *t*-test under the LCS setup replicated the original finding of a non-significant change, for a reasonable ρ = 0.50 correlation assumed between the two repeated measures. For higher values of ρ, however, the LCS *t*-test_P_ models began to indicate significant changes for ρ larger than.70; the LCS model with SF/M consistently yielded slightly lower *p*-values than the *t*-test_P_, and showed significant changes, whereas the *t*-test_P_ did not, for ρ 's between 65 and 70.

## Conclusions and recommendations

We showed how the classical paired *t*-test can be replicated in a SEM manner using LCS models, and illustrated the difference in results when one assumes an unexplained correlation between initial scores and changes, as opposed to initial scores predicting the changes. Between-individual and between-group differences in latent changes can be explained by various predictors in simple and more complex dynamical LCS models, hence the *t*-test_P_ replicated here as a LCS model with self-SF/M is a first step in building better models of change.

We suggest that researchers perform paired *t*-tests both in their classical form, which assumes that change is simply correlated with initial levels, and in their more realistic form, where a path from initial level to the LCS is specified. Both these LCS models can be freely replicated by all interested with our input models posted online, using the free version 5 of the AMOS software (Arbuckle, 2007) and summary (or raw) data entered in Microsoft Excel. The LCS setup introduced here allows for models explaining the variability in changes, by predictors like prior levels or even prior changes in other variables (), and can be expanded to more complex models, like recovering latent classes for which the change model differs markedly. One can model in other words heterogeneity in changes or of effects (with mixture LCS models, see AMOS example model online).

Any comparative tests of changes, as commonly performed for instance in Comparative Effectiveness Research (CER, Sox and Greenfield, 2009), can benefit from this accommodation of more flexible models supported by data. CER studies evaluate the success of interventions implemented in different conditions by comparing changes in different groups, like treatment and matched control, or different adaptations of the same intervention. In such multiple-group LCS models, the self-SF/M paths will likely differ also by group or condition, so there is increased chance of misspecification, because models assuming them to be equal or different will fit data better/worse. Estimates of differences in changes between groups will therefore depend on the specific pattern of changes in each group, revealed by the best fitting models. More research seems to be needed to see how the LCS with self-SF/M performs against the simple structural modeling extensions of the classical paired *t*-test.
